# Resting-State and Task-Based Functional Brain Connectivity in Developmental Dyslexia

**DOI:** 10.1093/cercor/bhu184

**Published:** 2014-08-28

**Authors:** Matthias Schurz, Heinz Wimmer, Fabio Richlan, Philipp Ludersdorfer, Johannes Klackl, Martin Kronbichler

**Affiliations:** 1 Centre for Cognitive Neuroscience; 2 Department of Psychology, University of Salzburg, 5020 Salzburg, Austria; 3 Neuroscience Institute, Christian Doppler Clinic, Paracelsus Medical University, Ignaz-Harrer-Str. 79, 5020 Salzburg, Austria

**Keywords:** dyslexia, fMRI, functional connectivity, reading, resting state, seed-voxel correlation mapping

## Abstract

Reading requires the interaction between multiple cognitive processes situated in distant brain areas. This makes the study of functional brain connectivity highly relevant for understanding developmental dyslexia. We used seed-voxel correlation mapping to analyse connectivity in a left-hemispheric network for task-based and resting-state fMRI data. Our main finding was reduced connectivity in dyslexic readers between left posterior temporal areas (fusiform, inferior temporal, middle temporal, superior temporal) and the left inferior frontal gyrus. Reduced connectivity in these networks was consistently present for 2 reading-related tasks and for the resting state, showing a permanent disruption which is also present in the absence of explicit task demands and potential group differences in performance. Furthermore, we found that connectivity between multiple reading-related areas and areas of the default mode network, in particular the precuneus, was stronger in dyslexic compared with nonimpaired readers.

## Introduction

Developmental dyslexia is a persistent difficulty in learning to read despite adequate intelligence and schooling; it has high prevalence and a familial and genetic risk (e.g., Gabrieli 2009).

Brain imaging research has found that developmental dyslexia is associated with reduced local brain activation during reading-related tasks in a left-hemispheric network. Based on 2 meta-analyses of imaging studies (Richlan et al. 2009, 2011), one of us concluded that developmental dyslexia is mainly linked to 3 brain areas: 1) the left posterior temporal cortex, 2) the left inferior parietal lobule (IPL), and 3) the left inferior frontal gyrus (IFG) (Richlan 2012).

As reading requires the cooperation between these distant brain areas, studying functional brain connectivity provides insights about the integration of neurocognitive processes in reading and dyslexia. A number of studies looked at task-based functional connectivity in dyslexia and analyzed temporal correlations between blood oxygenation level–dependent (BOLD) signal time series. The main finding in 2 of these studies (Shaywitz et al. 2003; van der Mark et al. 2011) was that dyslexic readers showed reduced functional connectivity between the left occipito-temporal cortex and the left IFG. This finding is of theoretical relevance because it reflects the linkage between visual-orthographic representations stored in the left occipito-temporal cortex and phonological and semantic word representations processed by the left IFG (Jobard et al. 2003). However, 2 other task-based connectivity studies could not find a group difference regarding the functional integration of these areas (Stanberry et al. 2006; Richards and Berninger 2008).

A potential limitation of task-based connectivity studies is that different reading tasks may produce different functional connectivity patterns, and thus, different dyslexic deficits. As recognized by Koyama et al. (2010), task-based functional connectivity research lacks consensus regarding the “optimal” task to characterize neural networks underlying reading and dyslexia. A possible solution is to study resting-state functional connectivity. At rest, low-frequency (<0.1 Hz) fluctuations in the BOLD signal are temporally correlated between functionally related brain areas (e.g., Fox and Raichle 2007). Resting-state data are obtained without any explicit task requirements, and therefore cannot be influenced by particular task demands. In addition, group differences in resting-state data cannot be linked to differences in task performance or processing strategies. Resting-state studies with nonimpaired readers found that reading-related brain areas are specifically linked to each other, forming positively correlated reading networks at rest (Koyama et al. 2010, 2011; see also Zhao et al. 2011; Tomasi and Volkow 2012; Vogel et al. 2012).

Although resting-state connectivity is a promising tool for brain research on dyslexia, little evidence from this approach exists. Farris et al. (2011) did a small pilot study with 5 dyslexic children and found reduced interhemispheric connectivity in dyslexic readers between left and right inferior frontal gyri. Recently, Koyama et al. (2013) studied resting-state connectivity in a group of dyslexic readers for left-hemispheric reading-related areas. The authors found no group differences in connectivity between many typically reading-related areas. What they did find was reduced resting-state connectivity between the left intraparietal sulcus and the left middle frontal gyrus, which was linked to a dysfunction of the frontoparietal dorsal attention network.

Put together, the available evidence does not show any deficits in dyslexic brain connectivity common to reading-related tasks and the resting state. This is surprising because resting-state connectivity is assumed to reflect a history of consistent and repeated co-activations of areas (Dosenbach et al. 2007; Fair et al. 2007). A recent study with nonimpaired participants compared resting-state with task-based functional connectivity patterns for 4 different cognitive tasks (Mennes et al. 2013). Correspondence between resting-state and task-based functional connectivity was found in parts of the frontoparietal control network and the default mode network. In contrast, for sensory cortices, which are relevant for studying dyslexia, correspondence was rather low. It was assumed that sensory areas show a relatively flexible and task-adaptive profile of functional interactions with other brain areas.

The present study examined both resting-state and task-based functional connectivity in the same group of dyslexic readers. To study task-based functional connectivity, we used 2 different reading-related tasks: silent reading and a phonological lexical decision task. The present study focused on brain areas typically underactivated in dyslexic readers. We performed correlation mapping for seed voxels within the 3 main areas linked to dyslexia (Richlan 2012): the left posterior temporal cortex, the left IPL, and the left IFG. With this approach, we looked for correspondences between dyslexic brain connectivity deficits for reading-related tasks and the resting state.

## Materials and Methods

### Participants

Fifteen German-speaking dyslexic adolescents and young adults (age range: 16–20 years) and 14 age-matched nonimpaired readers participated in the present study. Due to technical problems and image artifacts, resting-state scans are not available for 3 nonimpaired participants. For one dyslexic participant, we excluded the data for the phonological lexical decision task from our analysis, because head movements were too severe for a correction (140 of 146 scans were affected in one session). All participants were male, right-handed, and had normal or corrected-to-normal vision. The study was approved by the ethics committee of the University of Salzburg (Ethikkommission der Universität Salzburg). Participants gave written informed consent and were paid for their participation.

Group assignment was based on performance on a reading speed test in paper-and-pencil format. It presented a list of sentences from which as many as possible had to be marked as correct (making sense) or incorrect within 3 min. The content of these sentences was simple as the main aim of the test was to allow a quick assessment of reading speed impairments. Example items are “Dolphins and whales live in the sea,” or “Basketball can be played only during winter.” The format of this test corresponds to the reading fluency subtest of the Woodcock-Johnson III (WJ III) Test of Cognitive Abilities (COG; Woodcock et al. 2001). The test score was number of correctly marked sentences. In studies assessing the validity of similar published tests for school children, correlations between sentence reading scores and reading aloud performance on subtests of our Salzburger Lese- und Rechtschreibtest (Landerl et al. 1997) ranged from 0.76 to 0.81.

Performance on the reading speed test was transformed into a reading quotient based on a preliminary norm sample of about 300 adolescents and young adults. The reading quotient was scaled like the IQ score, *M* = 100, standard deviation (SD) = 15. Participants were assigned to the dyslexic group if their reading quotient was below 75, corresponding to percentile 5. All the nonimpaired readers had a reading quotient above 95, corresponding to percentile 37. Thus, reading performance of the best dyslexic reader and the worst nonimpaired reader differed in more than 1 SD. Nonimpaired readers had a mean reading quotient of 106, whereas dyslexic readers had a mean reading quotient of 63. The close to perfect accuracy of the dyslexic sample in evaluating the sentences rules out that their low test scores may reflect an accuracy problem. Slow reading speed in the absence of an accuracy problem is also evident from the additional reading measures in Table 1 which characterize reading aloud lists of words and pseudowords with increasing difficulty (time limit: 1 min).

**Table 1:** Means and standard deviations of participant characteristics

	Nonimpaired (*N* = 14)	Dyslexic readers (*N* = 15)	*t* (27)
Age (years)	17.77 (1.12)	18.28 (1.13)	0.51
Sentence reading			
Accuracy (% correct)	98.97 (0.62)	95.68 (3.81)	–
Speed (sentences correct/3 min)	56.21 (5.31)	25.73 (6.58)	13.67***
Corresponding reading quotient	106.57 (7.53)	63.34 (9.33)	
Word reading			
Accuracy (% correct)	99.86 (0.36)	97.33 (2.19)	–
Speed (items/min)	125.21 (11.60)	72.20 (22.35)	7.72***
Nonword reading			
Accuracy (% correct)	98.88 (1.54)	94.96 (5.97)	–
Speed (items/min)	83.36 (14.78)	41.00 (14.58)	7.49***
Verbal IQ			
Vocabulary	118.92 (9.84)	103.67 (10.08)	4.12***
Similarities	114.29 (9.38)	106.67 (10.47)	2.06*
Digit span	102.86 (11.88)	92.33 (11.32)	2.44*
Performance IQ			
Block design	109.29 (5.84)	112.00 (12.79)	0.73
Visual puzzles	106.79 (14.89)	111.33 (14.70)	0.83
Coding	106.42 (10.99)	96.33 (13.16)	2.23*
Silent reading task (in scanner)			
Accuracy (% correct)	99.27 (1.43)	96.86 (3.03)	–
Reaction time (ms/item)	877.13 (195.90)	955.34 (124.61)	1.26
Phonological lexical decision task (in scanner)			
Accuracy (% correct)	90.34 (5.40)	81.41 (8.58)	3.33**
Reaction time (ms/item)	957.48 (147.13)	1272.57 (283.03)	3.72**
Rapid automatized naming objects			
1-syllabic items (syl/min)	94.61 (19.08)	82.85 (16.82)	1.76
3-syllabic items (syl/min)	212.79 (47.42)	173.87 (32.66)	2.59*
All items (syl/min)	153.70 (31.59)	128.35 (23.81)	2.45*

Statistically reliable group differences are indicated by asterisks.

*P < 0.05, **P < 0.01, ***P < 0.001.

The specificity of the reading problem was ascertained by a nonverbal IQ score in the normal range (i.e., at least 90). Nonverbal IQ was measured by 3 subtests (Block Design, Visual Puzzles, and Coding) of the German adaptation (Tewes 1991) of the Wechsler Adult Intelligence Scale-Revised (WAIS-R). In addition, a rapid automatized naming (RAN) task after Denckla and Rudel (1976) was administered. We presented 2 lists of one- and three-syllabic object pictures, and assessed the number of articulated syllables per minute.

### Reading-Related Tasks

**Figure 1.**
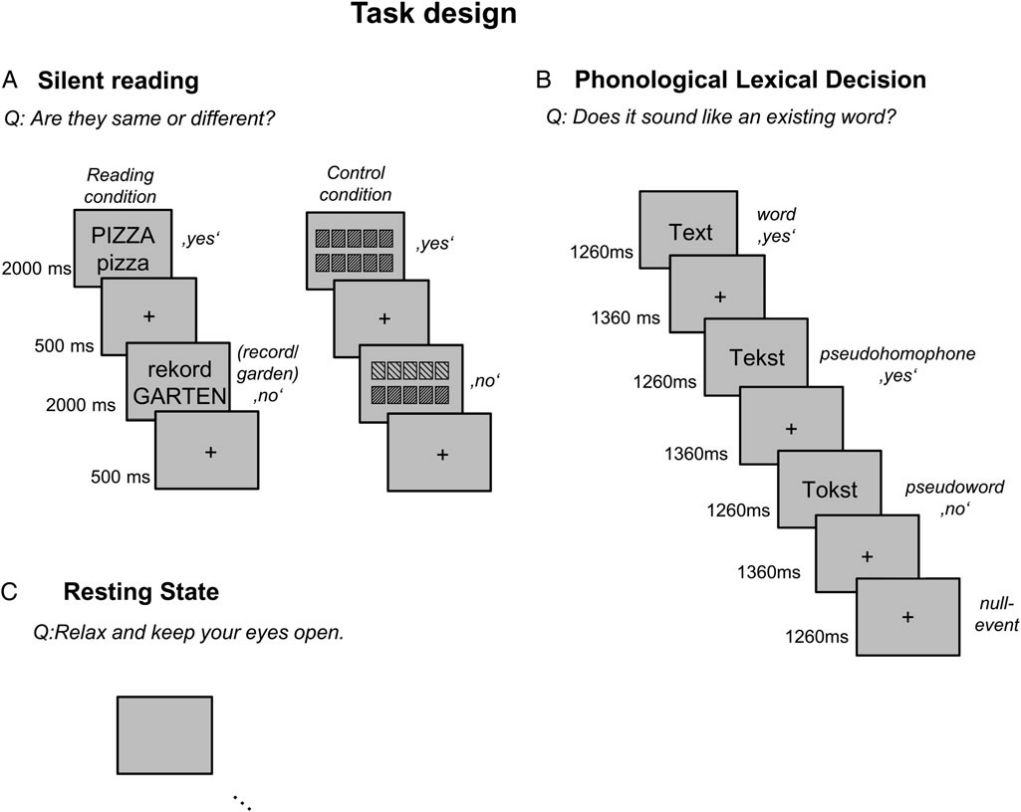
Illustration of the trials presented in the reading-related tasks and during rest. (*A*) Trials from the reading condition (left) and the control condition (right) of the silent reading task. (*B*) Trials for words, pseudohomophones and pseudowords, as well as a null event in the phonological lexical decision task. (*C*) During rest, a blank screen was presented, and participants were asked to relax and keep their eyes open.

In the silent reading task, participants saw either 2 words or 2 checkerboard-like images vertically aligned on the screen (see Fig. 1 for examples). Participants had to decide whether the 2 words were the same (irrespective of letter case) or whether the 2 checkerboard images were visually identical. Responses were given by button press. All presented words were of high frequency and consisted of 5 or 6 letters. A total of 30 word pairs and 30 checkerboard image pairs were presented in a block design (5 pairs were presented within 1 block of 17.5 s duration).

In the phonological lexical decision task, we presented words, pseudohomophones, and pseudowords (90 items each) and asked the participants to evaluate whether an item corresponds to an existing word (i.e., “Does xxx sound like an existing word?”). The same task was used in previous studies of nonimpaired (Kronbichler et al. 2007; Bruno et al. 2008; Schurz et al. 2010, 2014) and dyslexic readers (van der Mark et al. 2009, 2011; Wimmer et al. 2010; Richlan et al. 2010). In the present version of the task, items consisted of at least 3 and up to 10 letters. Examples are given in Figure 1. Further details on the stimuli can be found in Schurz et al. (2010). Responses were given by button press. A fast event-related design including null events was used. Each item was presented for 1260 ms with an interstimulus interval of 1360 ms during which a fixation cross was shown.

Table 1 reports the overall in-scanner behavioral performance of dyslexic and nonimpaired readers. For the silent reading task, there were no differences in accuracy and reaction times between nonimpaired and dyslexic readers. For the phonological lexical decision task, the number of errors made by dyslexic readers was nearly twice as high as in nonimpaired readers. In particular, dyslexic readers made more errors than nonimpaired readers for pseudohomophones and pseudowords. Average response latencies for the phonological lexical decision were prolonged by about 300 ms in dyslexic compared with nonimpaired readers. A stimulus-based analysis of the error rates and reaction times for the present data can be found in Richlan et al. (2010). This previous publication also reports results from the analysis of local brain activity for the phonological lexical decision task in both groups.

### Resting State

A high-resolution structural MR image was acquired (for ∼5 min) immediately before the resting-state session. For the rest period of 4 min, participants were asked to relax and keep their eyes open.

### Image Acquisition and Preprocessing

Data were obtained with a Philips Gyroscan NT 1.5 Tesla scanner (Philips Medical Systems, Inc., Maastricht, the Netherlands). The same sequence was used for the reading-related tasks and for the resting session: Functional images sensitive to BOLD contrast were acquired with a *T*_2_*-weighted gradient echo EPI sequence (TR 2200 ms, TE 45 ms, matrix-size 64 × 64 mm, field-of-view 220 mm, flip angle 90°). Twenty-five slices with a thickness of 5 mm and a slice gap of 0.7 mm were acquired in a sequential order (descending). Data from the silent reading task were recorded in 1 session with 101 scans, data from the phonological lexical decision task were recorded in 3 sessions with 146 scans per session, and resting-state data were recorded in 1 session with 110 scans. In addition, a high-resolution (1 × 1 × 1.2 mm) structural scan was acquired from each participant with a *T*_1_-weighted MPRAGE sequence. For preprocessing and parts of statistical data analysis, SPM8 software was used (http://www.fil.ion.ucl.ac.uk/spm) running in a MATLAB 7.6 environment (Mathworks, Inc., Sherbon, MA, USA). We applied the same basic preprocessing steps for all 3 functional datasets. Functional images were realigned and unwarped. After realignment, we controlled all functional images for head movement artifacts. Recent studies (e.g., Power et al. 2012; Van Dijk et al. 2012) showed that functional connectivity results can be severely affected by head movements, even if standard post hoc motion correction methods are applied. We used a method for detection and repair of bad volumes implemented in the ArtRepair toolbox (http://cibsr.stanford.edu/tools/human-brain-project/artrepair-software.html; Mazaika et al. 2009). Two measures were used to detect bad volumes: 1) % variation in the global average BOLD signal from scan to scan and 2) frame-wise displacement, reflecting the sum all head movements from scan to scan (calculated from realignment parameters). For these measures, we used the software default cutoff score: 1) 1.5% variation in the global signal and 2) 0.5 mm/TR. If any cutoff was exceeded for a volume, it was automatically repaired (i.e., replaced via interpolation). After our artifact detection, we co-registered the functional data to the high-resolution structural image (data of the phonological lexical decision task were additionally slice time corrected). The structural image was normalized to the MNI T1 template image (via segmentation), and the resulting parameters were used for normalization of the functional images, which were resampled to isotropic 3 × 3 × 3 mm voxels and smoothed with an 8-mm FWHM Gaussian kernel.

### Analysis of Functional Connectivity

Functional connectivity was analyzed with seed-voxel correlation mapping with the CONN-fMRI toolbox 13.i for SPM (http://www.nitrc.org/projects/conn; Whitfield-Gabrieli and Nieto-Castanon 2012). This method computes the temporal correlation between brain activity from a given area to all other areas using a General Linear Model approach. Resting-state data were band-pass filtered (0.008–0.09 Hz). For all data, we modeled nuisance covariates including cerebrospinal fluid and white-matter signals and their derivatives, following the CompCor strategy (Behzadi et al. 2007), as implemented in CONN. Activity within 6-mm spherical Regions of Interest (ROIs) were extracted and correlated with the activity in all other areas of the brain. ROIs were defined within the 3 main areas linked to dyslexia (Richlan 2012), and locations of seed regions were based on the coordinates of a quantitative meta-analysis (Richlan 2009). In the left posterior temporal cortex, we selected seed regions in the fusiform gyrus (FFG), *x* = −46, *y* = −50, *z* = −16; the inferior temporal gyrus (ITG), *x* = −52, *y* = −62, *z* = −8; the middle temporal gyrus (MTG), *x* = −60, *y* = −56, *z* = 2; and the superior temporal gyrus (STG), *x* = −52, *y* = −44, *z* = 20. In addition, we selected one seed region in the left IPL, *x* = −52, *y* = −46, *z* = 44, and one in the left IFG pars opercularis, *x* = −46, *y* = 16, *z* = 6.

Subject-specific contrast images reflecting standardized correlation coefficients were used for the second-level random-effects analysis in SPM. We computed one-sample *t*-tests on the correlation coefficients to determine positive and negative functional connectivity maps within groups. Group- and task-related differences in functional connectivity were computed in the context of an Analysis of Variance (ANOVA) (i.e., a full-factorial design). We used a set of standard *t*-contrasts (automatically generated by the SPM software for ANOVAs) to assess the main effect of group and the interaction between group and task. All results were thresholded at a voxel-wise *P* < 0.001 and a cluster extent *P* < 0.05 FWE corrected.

## Results

The central results of our ANOVA are the main effect of group (nonimpaired and dyslexic) and the interaction between group and task (rest, silent reading, and phonological lexical decision). To keep this report short, we only present these group-related effects. The main effect of task (across both groups) will not be reported since it is of little relevance for our dyslexia-focused research question. Connectivity patterns within groups, as well as task-specific group differences, are shown in Supplementary Figs 1–6.

### Occipito-Temporal Seed Areas: Left Fusiform Gyrus and Left Inferior Temporal Gyrus

**Figure 2.**
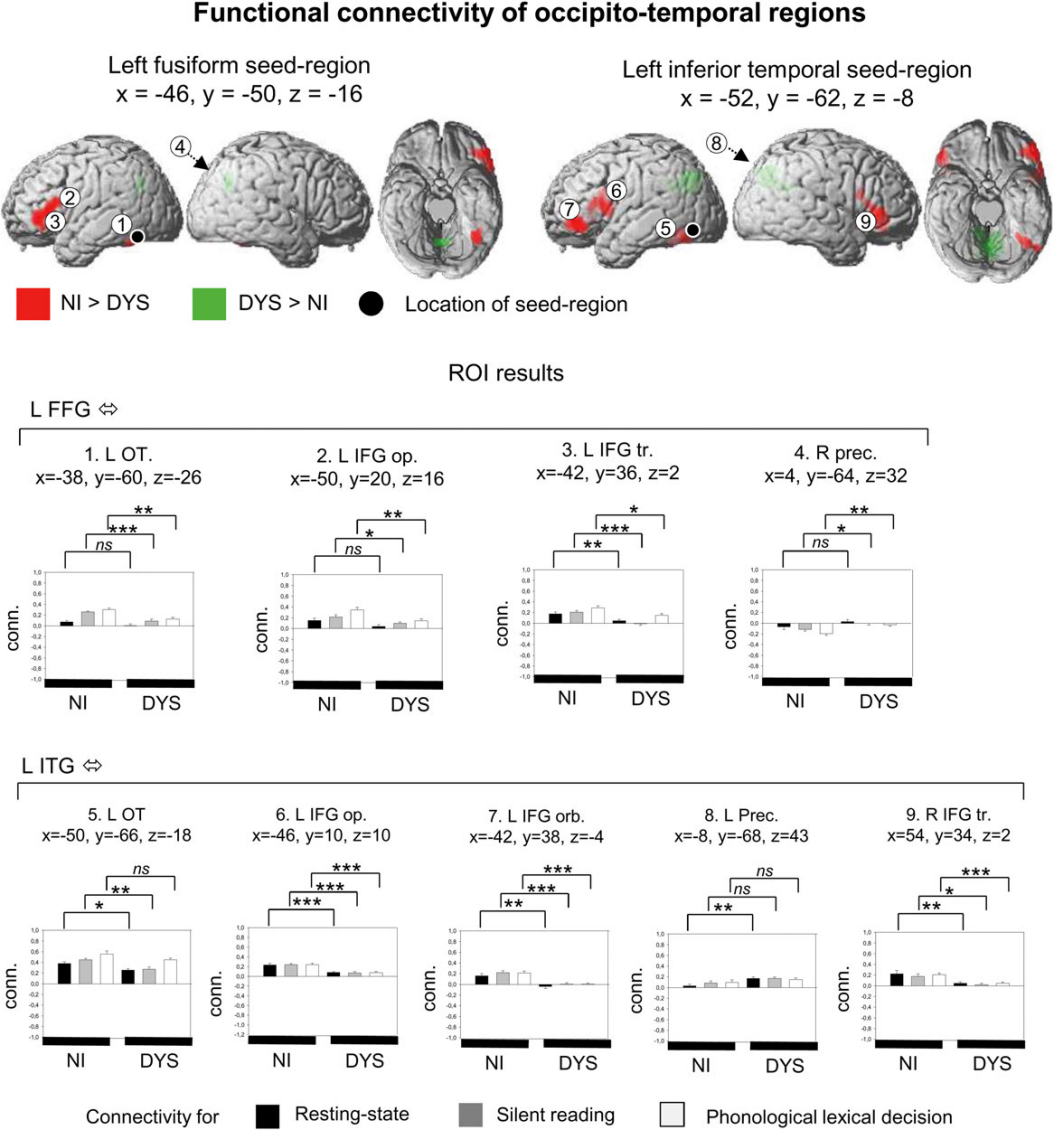
Functional connectivity for seed regions in the left fusiform gyrus and the left inferior temporal gyrus. Group differences are shown in terms of main effects, stronger activation for nonimpaired readers is shown in red, stronger activation for dyslexic readers is shown in green. Maps are shown at a voxel-wise threshold of *P* < 0.001 uncorrected and a cluster extent threshold of *P* < 0.05 FWE-corrected. Brain connectivity estimates are shown for regions of interest. Connectivity values correspond to standardized correlation coefficients. *P < 0.05,**P < 0.01.

Results on connectivity of the left FFG and the left ITG seed regions are shown in Figure 2 and in Table 2. Main effects were found as stronger connectivity in nonimpaired compared with dyslexic readers between left FFG and adjacent areas of left FFG/cerebellum, and left FFG and left IFG pars triangularis/opercularis. ROI analyses showed that the group difference in connectivity between left FFG and adjacent areas of left FFG fusiform, and between left FFG and left IFG pars opercularis connectivity were present for silent reading and phonological lexical decision (*t*s > 2.2, *P*s < 0.05) but not found during rest. The group difference in left FFG–left IFG triangularis connectivity was present for both tasks and rest (*t*s > 2.7, *P*s < 0.05). Conversely, we found a main effect showing stronger connectivity in dyslexic compared with nonimpaired readers between left FFG and right precuneus. ROI analysis found this difference significant for silent reading and phonological lexical decision (*t*s > 2.6, *P*s < 0.05) but not during rest. Our whole-brain analysis found no interactions between group and task (silent reading, phonological lexical decision, and rest) on connectivity of the left FFG.

**Table 2:** Significant group main effects for each seed region

Seed	Correlated area	MNI			*t*	vx
		*X*	*y*	*z*		
	Nonimpaired > dyslexic					
L fusiform	L cereb./fusiform	−38	−60	−26	5.09	194
	L cereb.	−30	−52	−32	3.72	–
	L IFG triang.	−42	36	2	5.77	584
	L IFG triang.	−54	26	12	4.29	–
	L IFG operc./triang.	−50	20	16	3.79	–
L inf. temp.	L inf. occipit.	−50	−66	−18	4.44	249
	L fusiform	−38	−60	−12	4.35	–
	L IFG operc.	−46	10	10	7.02	436
	L IFG triang.	−56	26	4	3.34	–
	L IFG triang.	−42	38	−4	6.92	503
	L IFG orbit.	−42	42	−10	3.67	–
	R IFG triang.	54	34	2	5.20	381
	R IFG operc.	52	14	18	3.34	–
L mid. temp.	L IFG orbit.	−44	38	−6	4.88	590
	L temp. pole	−52	20	−12	4.53	–
	L temp. pole	−54	−10	−4	3.86	–
	R front. mid. orbit.	34	44	−10	4.82	303
	R IFG orbit.	52	40	−12	4.80	–
L sup. temp.	L IFG triang.	−46	34	0	5.44	293
	L IFG orbit.	−48	34	−4	4.66	–
L inf. parietal	L front. pole	−40	52	−6	5.98	721
	L IFG orbit.	−44	40	−2	5.65	–
	L IFG orbit.	−46	40	−12	5.00	–
	R IFG orbit.	46	42	−12	5.98	584
	R IFG orbit.	44	50	−10	5.23	–
	R mid. front.	38	64	4	4.93	–
	R inf. temp.	68	−46	−10	5.69	233
	R inf. temp.	62	−42	−20	4.45	–
	R cerebellum	26	−62	−36	4.94	185
	R cerebellum	34	−70	−44	4.11	–
	L cerebellum	−38	−68	−34	4.77	251
	L cerebellum	−34	−70	−48	3.96	–
	L cerebellum	−4	−82	−36	4.64	198
	R cerebellum	12	−78	−36	4.32	–
L IFG operc.	L mid. temp./pSTS	−52	−46	16	5.54	302
	L mid. temp.	−52	−50	4	3.79	−
	L suppl. motor area	0	14	58	4.89	172
	L suppl. motor area	−6	8	62	4.75	–
L fusiform	R precuneus	4	−64	32	4.01	249
	L precuneus	−4	−64	30	3.89	–
L inf. temp.	L precuneus	−8	−68	43	5.68	944
	L post. cing.	−8	−50	32	3.58	–
	R precuneus	12	−50	28	4.14	–
L mid. temp.	L calcarine	−18	−74	16	4.86	343
	L cuneus	−10	−82	24	4.30	–
	R cuneus	6	−78	30	4.76	–
L sup. temp.	–					
L inf. parietal	R fusiform/hippocampus	40	−6	−28	5.51	396
	R mid. temp.	52	4	−22	3.65	–
	L mid. front.	−24	40	28	5.13	229
	L sup. front.	−26	54	38	3.85	–
	L precuneus	−6	−58	42	5.05	860
	L post. cingulate	−12	−48	24	4.84	–
	Ant. cingulate	0	34	16	4.61	1101
	R front. med. orbit	6	42	−10	4.55	–
	R sup. front./mPFC	2	56	12	4.05	–
L IFG operc.	R insula/rol. operc.	42	−6	14	5.01	150
	R rol. operc.	48	−8	14	4.36	–
	R angular	60	−60	32	4.52	232
	R supramarginal	64	−40	34	3.38	–
	L inf. parietal	−50	−54	50	4.45	418
	L angular	−56	−56	36	4.24	–
	L inf. parietal	−56	−44	48	4.02	–

Voxel-level threshold of *P* < 0.001 and cluster extent *P* < 0.05 corrected.

For the left ITG seed area, main effects showing stronger connectivity in nonimpaired compared with dyslexic readers were found for the connections left ITG–left FFG, left ITG–left IFG pars opercularis, left ITG–left IFG pars orbitalis, and left ITG–right IFG pars triangularis. ROI analyses found that stronger connectivity for nonimpaired readers between left ITG and left FFG was present for rest and silent reading (*t*s > 2.7, *P*s < 0.05) but not for the phonological lexical decision. All connectivity group differences between left ITG and IFG (left and right IFG, triangularis, opercularis, and orbitalis) were present during both tasks and rest (*t*s > 2.7, *P*s < 0.05). On the other hand, we found stronger connectivity (main effect) in dyslexic compared with nonimpaired readers between left IFG and left precuneus. ROI analysis showed that this finding was only present during rest (*t* = 3.4, *P* < 0.01) but not during the tasks. No interactions between group and task were found for the left ITG.

### Left Temporo-Parietal Seed Areas: Left Superior Temporal Gyrus and Left Inferior Parietal Lobule

**Figure 3.**
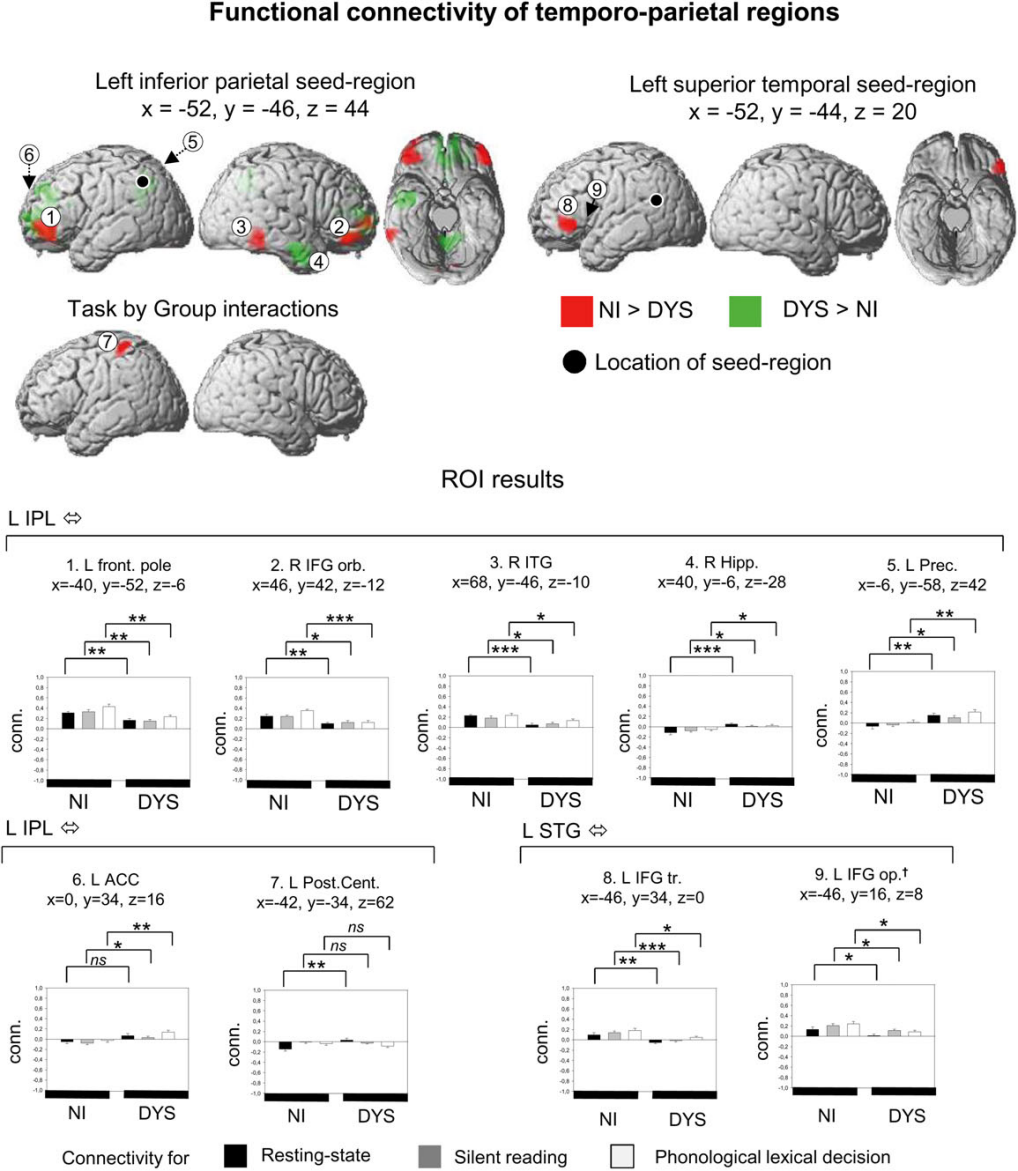
Functional connectivity for seed regions in the left inferior parietal lobule and the left posterior superior temporal gyrus. † area found at an uncorrected threshold of p < 0.001 and 20 voxel extent on the whole-brain level. All other details same as in Figure 2.

Results for the left STG and the left IPL seed areas are shown in Figure 3 and details are given in Table 2. For the left STG, we only found a main effect with stronger correlation in nonimpaired readers between left STG and left IFG pars triangularis. In addition, we checked for connectivity differences at a lower statistical threshold of *P* < 0.001 uncorrected and 20 voxel extent, with a focus on the classical “phonological loop” which is often characterized by IFG pars opercularis and posterior STG. We could find such a cluster in the left IFG pars opercularis (*x* = −46, *y* = −16, *z* = 8, 98 voxel extent) showing reduced connectivity to the left STG in dyslexic readers. ROI analysis showed that for both the left IFG pars opercularis and pars triangularis, group difference were present for both tasks and rest (*t*s > 2.0, *P*s < 0.05). No areas showing stronger connectivity in dyslexic compared with nonimpaired readers were found for left STG connectivity. Furthermore, no interactions between group and task were found.

For the left IPL seed, we found main effects showing stronger connectivity in nonimpaired readers for 6 connections, namely left IPL–frontal pole, left IPL–left IFG pars orbitalis, left IPL–right ITG, left IPL–right cerebellum, and left IPL–left cerebellum (2 distinct left cerebellar findings). ROI analyses found that connectivity between IPL and cortical areas (frontal pole, IFG, inferior temporal) was reduced in dyslexic readers for both tasks and rest (*t*s > 2.0, *P*s < 0.05). Connectivity between left IPL and cerebellar regions showed a mixed pattern. For left IPL–left cerebellum (*x* = −38, *y* = −68, *z* = −34), stronger connectivity in nonimpaired readers was only found at rest (*t* = 5.2, *P* < 0.001), but not during the tasks. For left IPL–right cerebellum, we found stronger connectivity for both tasks and rest (*t*s > 2.4, *P*s < 0.05). Group main effects in the opposite direction, namely stronger for dyslexic compared with nonimpaired readers were found between left IPL and right fusiform/hippocampus, left IPL and left middle frontal gyrus, left IPL and left precuneus, and left IPL and anterior cingulate gyrus. ROI analyses showed group difference for both tasks and rest (*t*s > 2.0, *P*s < 0.05) on the connections between left IPL and right hippocampus, and left IPL and left precuneus. For the connection between left IPL and left anterior cingulate, we only found group differences for the tasks (*t*s > 2.0, *P*s < 0.05) but not for rest.

Finally, we found an interaction between group and task on connectivity between left IPL and left postcentral gyrus. The postcentral area functionally connected to the left IPL had its peak at *x* = −42, *y* = −34, *z* = 62 and had 151 voxel extent. ROI analysis showed that here stronger connectivity was found in dyslexic readers, but only at rest (*t* = 3.5, *P* < 0.01) and not during the reading-related tasks.

### Left Temporal and Frontal Seed Areas: The Left Middle Temporal Gyrus and the Left Inferior Frontal Gyrus

**Figure 4.**
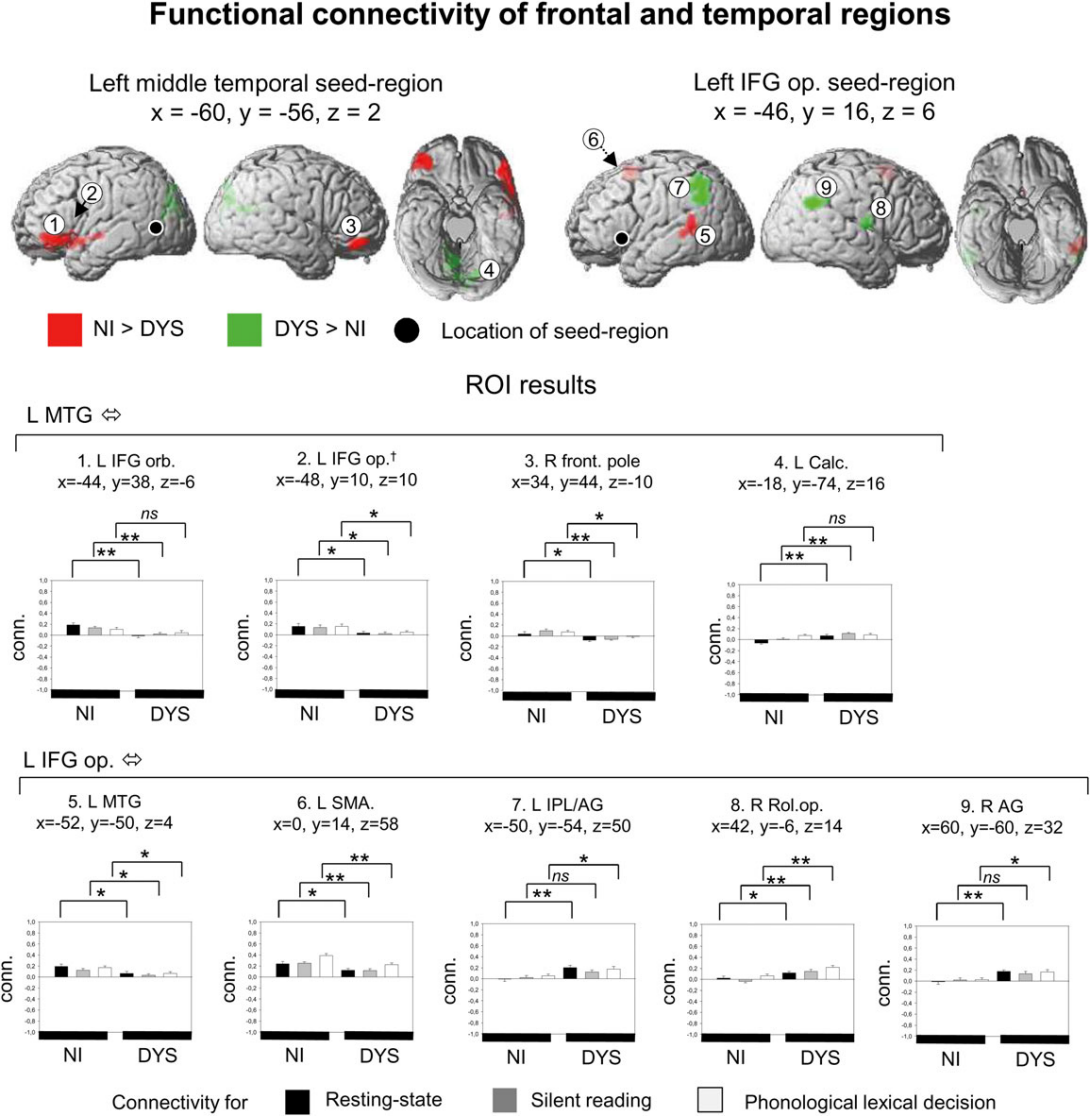
Functional connectivity for seed regions in the left posterior middle temporal gyrus and the left inferior frontal gyrus pars opercularis. † area found at an uncorrected threshold of p < 0.001 and 20 voxel extent on the whole-brain level. All other details same as in Figure 2.

Figure 4 shows results for left MTG and left IFG seed areas. Again, the details for connectivity clusters are given in Table 2. For the left MTG seed, we found group main effects in the form of stronger connectivity for nonimpaired readers between left MTG and left IFG pars orbitalis, and left MTG and right frontal pole. To check consistency with a finding from our IFG seed area (see below), we lowered the statistical threshold to *P* < 0.001 uncorrected and 20 voxel extent. Now, we also found stronger connectivity in dyslexic readers between left MTG and left IFG pars opercularis. For this connection, ROI analysis found stronger correlation for nonimpaired readers at rest and for silent reading (*t*s > 2.8, *P*s < 0.01), but not for phonological lexical decision. For connectivity between left MTG and left IFG pars opercularis, and left MTG and right frontal pole, group differences were present for both tasks and rest (*t*s > 2.1, *P*s < 0.05). We also found a group main effect in the opposite direction, that is, stronger connectivity for dyslexic compared with nonimpaired readers. This was found for the connection left MTG–left calcarine sulcus. ROI analysis showed that this group difference was present at rest and for silent reading (*t*s > 3.3, *P*s < 0.01), but not for the phonological lexical decision task.

For the left IFG seed, we found group main effects showing stronger connectivity for nonimpaired readers between left IFG and left MTG, and left IFG and left SMA. ROI analysis showed that, for both findings, group differences were present for both tasks and the resting state (*t*s > 2.2, *P*s < 0.05). On the other hand, we also found main effects that showed stronger connectivity in dyslexic readers, namely between left IFG and left IPL/angular, left IFG and right angular, and left IFG and right rolandic operculum. For connectivity between left IFG and bilateral IPL/angular, ROI analysis found only group differences at rest and for the phonological lexical decision task (*t*s > 2.0, *P*s < 0.05), but not for silent reading. For connectivity between left IFG and right rolandic operculum, stronger correlations for dyslexic readers were found for both tasks and at rest (*t*s > 2.3, *P*s < 0.05). We found no interaction between group and task on connectivity of the left IFG.

### Correlations Between Brain Connectivity and Behavior

We correlated brain connectivity (standardized seed-voxel correlation coefficients) with behavioral measures obtained outside the scanner, namely reading fluency (number of correctly marked sentence/3 min), RAN (number of articulated syllables/1 min; 1- and 3-syllabic items taken together) and verbal IQ (WAIS-R Vocabulary score, number of correct items). Brain–behavior correlations were calculated across groups. A complete list of brain–behavior correlations for all main findings is given in the Supplementary Materials. To keep it short, in Table 3, we focus on brain–behavior correlations between seed areas and left-hemispheric reading-related regions. Our brain–behavior correlation analyses implied multiple testing (25 tests), and therefore only highly significant findings (*P* < 0.001) reported in Table 3 correspond to significance after multiple comparison correction (Bonferroni). We found that brain connectivity between our occipito-temporal areas (FFG and ITG) and adjacent occipito-temporal areas, as well as inferior frontal areas was correlated to reading fluency and often also to verbal IQ. Brain connectivity between FFG and left IFG pars opercularis, and ITG and left IFG pars opercularis additionally showed correlations with naming speed. Brain connectivity between left MTG and frontal areas was mainly correlated to reading fluency, and brain connectivity between left STG and the IFG was mainly correlated to reading fluency and verbal IQ. Negative correlations between brain-connectivity and behavioral measures were found between left IFG and left IPL/angular gyrus. When calculating partial correlations controlling for the group factor, almost all correlations reported in Table 3 turned out to be nonsignificant, which suggests that the brain–behavior correlations were largely driven by group differences in behavior.

**Table 3:** Brain–behavior correlations (Pearson's *r*) for left-hemispheric reading-related connections

Connection	Reading	Naming	Verbal IQ
FFG ⇔ L Cereb./OT			
Rest	0.16	0.28	0.06
Silent reading	0.66***	0.42*	0.46*
Phon. Lex. Dec.	0.58**	0.37	0.71***^†^
FFG ⇔ L IFG op.			
Rest	0.50**^†^	0.35	0.39*
Silent reading	0.57**^†^	0.48**	0.49**
Phon. Lex. Dec.	0.66***^†^	0.50**	0.53**
FFG ⇔ L IFG tr.			
Rest	0.55**	0.27	0.53**
Silent reading	0.62**	0.28	0.40*
Phon. Lex. Dec.	0.48**	0.26	0.49**
ITG ⇔ L OT			
Rest	0.43*	0.06	0.33
Silent reading	0.65***	0.47*	0.53**
Phon. Lex. Dec.	0.28	0.09	0.25
ITG ⇔ L IFG op.			
Rest	0.56**	0.48*	0.29
Silent reading	0.64***	0.57**^†^	0.47*
Phon. Lex. Dec.	0.65***	0.38*	0.44*
ITG ⇔ L IFG tr.			
Rest	0.54**	0.11	0.42*
Silent reading	0.57**	0.21	0.35
Phon. Lex. Dec.	0.59**	0.15	0.43*
MTG ⇔ L IFG orb.			
Rest	59**	0.18	0.19
Silent reading	0.54**	0.31	0.16
Phon. Lex. Dec.	0.18	0.22	−0.23
MTG ⇔ L IFG op.			
Rest	0.41*	0.15	0.38
Silent reading	0.45*	0.34	0.37*
Phon. Lex. Dec.	0.35	0.10	0.20
STG ⇔ L IFG tr.			
Rest	0.55**	0.31	0.57**
Silent reading	0.65***	0.33	0.49**
Phon. Lex. Dec.	0.47*	−0.13	0.20
STG ⇔ L IFG op.			
Rest	0.55**	0.55**	0.51**^†^
Silent reading	0.44*	0.10	0.38*
Phon. Lex. Dec.	0.47*	0.02	0.21*
IFG ⇔ L MTG			
Rest	0.45*	0.22	0.33
Silent reading	0.33	−0.06	0.07
Phon. Lex. Dec.	0.30	0.09	−0.05^†^
IFG ⇔ SMA			
Rest	0.37	0.19	0.38
Silent reading	0.48**	0.39*	0.29
Phon. Lex. Dec.	0.48*	0.20	0.44*
IFG ⇔ L IPL/AG			
Rest	−0.55**	−0.51**	−0.47*
Silent reading	−0.31	−0.29	−0.32
Phon. Lex. Dec.	−0.39*	−0.28	−0.11

*P < 0.05, **P < 0.01, ***P < 0.001, ^†^*P* < 0.05 in partial correlation controlling for group factor.

## Discussion

We analyzed task-based and resting-state functional connectivity for reading-related brain areas in nonimpaired and dyslexic readers. Our main finding was reduced connectivity in dyslexic readers between left posterior temporal areas (FFG, ITG, MTG, and STG) and the IFG. Findings of reduced connectivity implicated both the IFG pars opercularis and the IFG pars triangularis for all 4 left posterior temporal areas. Interestingly, disrupted connectivity to the IFG pars opercularis was most pronounced for ventral left posterior temporal areas (FFG and ITG). Almost all of these findings were consistently present for 2 reading-related tasks and for the resting state. This implies that dyslexic readers show a permanent disruption in the functional integration between frontal and temporal brain areas, which is also present in the absence of explicit task demands and potential group differences in performance. For the left IPL, dyslexic readers showed reduced connectivity to the left ventral middle frontal gyrus, which may be linked to executive control rather than classical language functions. On the other hand, we found that dyslexic readers show stronger connectivity between multiple reading-related areas (FFG, ITG, MTG, IPL, and IFG) and areas linked to the default mode network, in particular the precuneus.

### Main Findings: Connectivity Differences in Fronto-Temporal Reading Networks

The main finding of the present study was that dyslexic readers showed reduced functional connectivity between the left posterior temporal areas and the IFG. In particular, for the left FFG and the left ITG, we found reduced connectivity to both IFG pars triangularis and IFG pars opercularis at a statistical threshold corrected for multiple comparisons. For the MTG and STG, only findings of reduced connectivity implicating the IFG pars triangularis survived the correction for multiple comparisons, whereas findings implicating the left IFG pars opercularis remained as a tendency (*P* < 0.001 uncorr.). It is also noteworthy that we did not find any other alterations in functional connectivity between left posterior temporal areas, for example the FFG, STG, and IPL.

Our findings are consistent with task-based functional connectivity research (Shaywitz et al. 2003; van der Mark et al. 2011; Finn et al. 2013) that found reduced coupling between the left occipito-temporal areas and the left IFG. In particular, a recent study (Finn et al. 2013) used an unbiased whole-brain parcellation method which allows identifying disrupted connectivity networks fMRI data without a priori definition of a seed area. In line with the present fining, the authors found reduced connectivity between the left FFG and the left IFG. The present findings go one step beyond previous ones as they show that disruptions in functional connectivity generalize across different reading-related tasks and can also be found at rest. Diffusion-tensor imaging studies found reduced white-matter integrity in dyslexic readers in left temporo-parietal (e.g., Klingberg et al. 2000; Beaulieu et al. 2005; Deutsch et al. 2005) and left frontal areas (e.g., Steinbrink et al. 2008; Rimrodt et al. 2010; Gebauer et al. 2012) which were often linked to a structural deficit of the left superior longitudinal fasciculus (SLF), and in particular, the left arcuate fasciculus (for a meta-analysis, see Vandermosten et al. 2012). Anatomically, the left SLF connects areas of the left posterior temporal lobe and IPL to areas near the IFG pars opercularis (e.g., Catani et al. 2005; Glasser and Rilling 2008). Our most ventral seed area in the FFG might not be directly linked to the SLF. In this respect, it is of interest that we observed a functional coupling between the left FFG and left ITG in nonimpaired but not dyslexic readers, which could explain how information is passed indirectly from the FFG to the left IFG pars opercularis in nonimpaired reading.

Recent MEG-based research found that occipito-temporal areas receive top-down signals from IFG already during the first 200 ms of visual word processing (Woodhead et al. 2013, 2014). Interestingly, McCrory et al. (2005) found that dyslexic readers exhibited reduced local brain activation in the occipito-temporal cortex not only for word reading but also for picture naming. This indicates an impairment of a more general function which implies the left occipito-temporal cortex, namely the integration between visual and verbal information (see also Price and Devlin 2003, 2011; Devlin et al. 2006; Hellyer et al. 2011; Kherif et al. 2011). We found support for this hypothesis by showing permanently reduced connectivity between left occipito-temporal areas and the left IFG. This offers a neurobiological explanation why dyslexic readers not only show speed impairments for reading but also for rapid naming of objects, numbers and colors (see Wimmer and Schurz 2010 for a review). Longitudinal studies show that rapid naming speed in Kindergarten is an important predictor of later reading problems in school (e.g., Wimmer et al. 2000; Lervag and Hulme 2009). Notably, we found that brain connectivity between occipito-temporal areas and IFG pars opercularis showed correlations not only with reading fluency but also with rapid naming speed measured outside the scanner.

For the MTG and STG, we found similar patterns of dyslexic connectivity differences as for the FFG and ITG. Connectivity differences of the MTG and the STG were less strong with respect to the IFG pars opercularis. However, when only looking at our nonimpaired readers, we observed that connectivity between left STG and left IFG pars opercularis was stronger than that between left STG and IFG pars triangularis—which is consistent with the classical concept of the Wernicke's Area − Broca' Area circuit for language comprehension (see Tomasi and Volkow 2012 for a large-scale validation with resting-state fMRI). A recent meta-analysis (Richlan et al. 2011) showed that dyslexic underactivation in the left posterior STG is consistently found only in adults but not in children, which questions the traditional view that dyslexic readers suffer from a primary dysfunction of the temporo-parietal cortex (centered on the pSTG) involved in controlled grapho-phonological word processing (e.g., Pugh et al. 2000; McCandliss and Noble 2003). In this regard, we note that a recent neuroimaging meta-analysis found that phonological processing of speech (phonemes and words) is located in mid and anterior STG, which challenges the classical assumption that auditory word-form recognition is localized in posterior STG/STS (DeWitt and Rauschecker 2012). When taking the IFG pars opercularis itself as the seed area, we found reduced dyslexic connectivity with a more anterior portion of MTG/STS. In line with our findings, Boets et al. (2013) recently showed with multivoxel pattern analysis that phonetic representations of speech sounds are stored in primary and secondary auditory cortices. For dyslexic readers, these phonetic representations were intact. However, based on structural and functional connectivity measures, the authors found that the access to these representations by the IFG was impaired.

### Other Findings: Connectivity Differences in Executive and Default Mode Networks

For our seed area in the IPL, we found reduced connectivity in dyslexic readers with the left middle frontal areas and the frontal pole. These areas are not typically linked to the reading network. A recent meta-analysis (Niendam et al. 2012) found the middle frontal gyrus to be part of a superordinate cognitive control network which implicated in various executive functions (e.g., flexibility, inhibition, and working memory). Importantly, this cognitive control network also included parts of the left IPL. Likewise, recent review of dyslexic brain dysfunctions (Shaywitz and Shaywitz 2008) linked the IPL to general attentional mechanisms, which may interact with reading processes.

Besides findings of stronger connectivity in nonimpaired compared with dyslexic readers, we also found the opposite pattern for some of our seed areas. Connectivity between multiple reading-related areas (FFG, ITG, MTG, and IPL) and posterior cortical midline areas (precuneus/cuneus) were stronger in dyslexic compared with nonimpaired readers. These group differences were mainly driven by stronger positive correlations in dyslexic reader.

Another finding of stronger connectivity in dyslexic readers was linked to IFG pars opercularis–left IPL/angular gyrus and IFG pars opercularis–right angular gyrus. For the interpretation of these findings, we consider that recent (ICA-based) resting-state functional connectivity research found 3 distinct and functionally segregated connectivity networks that involve different parts of the temporo-parietal cortex: 1) the default mode network, 2) the frontoparietal network, and 3) the dorsal attention network (e.g., Fox et al. 2005; Vincent et al. 2008; Andrews-Hanna et al. 2010). Anatomically, the dorsal attention and the frontoparietal network subsume most reading-related regions. The default mode network covers cortical midline areas and, interestingly, also posterior parts of the temporo-parietal cortex including the angular gyrus. Meta-analyses of local brain activity show robust task-related deactivations in the left angular gyrus (Spreng et al. 2008; Laird et al. 2009), and a specific functional role of the left angular gyrus for reading was questioned in literature reviews (Price 2000; Jobard et al. 2003). We speculate that our findings could—at least in part—correspond to these angular regions linked with the default mode network.

### Orthography-Related Considerations

In our study, the criterion for assigning participants to the dyslexia group was a difference in reading fluency. This is because we tested readers of German, which has high regularity in grapheme-phoneme mappings (shallow orthography). In German, reading accuracy reaches ceiling levels very rapidly in beginning readers (Wimmer et al. 1998; Vaessen and Blomert 2010), and dyslexic readers usually have little problems with accurate reading (Klicpera and Schabmann 1993; Wimmer et al. 1998; Zoccolotti et al. 1999). These behavioral patterns stand in contrast to what is found for deep orthographies like English, where dyslexic readers suffer from both inaccurate and disfluent reading. To study these behavioral differences on the neural level, Paulesu et al. (2001) compared brain activation for word- and pseudoword reading between dyslexic readers of shallow versus deep orthographies. No orthography-related differences were found in dyslexic brain function, which was taken as evidence for a universal neurocognitive basis (see Richlan 2014 for discussion). Based on the results of the present study, we suggest for future studies to look at the impact of orthographic depth on functional brain connectivity. Although our data show some commonalities with results from connectivity studies in deep orthographies (i.e., English, see Finn et al. 2013), a direct comparison in future studies would be of high interest. For example, identical local brain dysfunctions could be embedded in different network interactions in different orthographies.

### Limitations

#### Sample Size

The sample size of our study (*n* = 29) is limited due to practical reasons. It is comparable with that of other functional imaging studies on dyslexia (Schulz et al. 2009, *n* = 30; Booth et al. 2007, *n* = 32; Cao et al. 2006, *n* = 28; Hoeft et al. 2006, *n* = 30; Blau et al. 2009, *n* = 26; Brambati et al. 2006, *n* = 24; Rumsey et al. 1997, *n* = 31). Recent work (Button et al. 2013) showed that neuroimaging studies need larger sample sizes to increase the power and reliability of findings. This clearly also applies to our study. Still, a positive feature of our study is that our approach was in parts confirmatory, that is, we tested whether dyslexic abnormalities found in previous studies for task-based functional connectivity (Shaywitz et al. 2003; van der Mark et al. 2011; see also Finn et al. 2013) generalize across reading-related tasks and the resting state. Our data support this hypothesis. Moreover, our main results regarding fronto-temporal networks are consistent with recent findings based on a large sample of nonimpaired and dyslexic readers (*n* = 104, Finn et al. 2013).

#### Verbal IQ

It must be noted that we found significant group differences for subtests of our IQ assessment. First, we found a group difference for the Coding subtest of performance IQ. Second, we found group differences for all verbal IQ subtests, in particular for Vocabulary. Still, dyslexic performance on all subtests fell within the average range. On a standard IQ scale (*M* = 100, SD = 15), dyslexic mean scores were 96 (Coding), 104 (Vocabulary), 107 (Similarities), and 92 (Digit Span). Although IQ scores on these subtests were higher for nonimpaired readers, they also mostly fell within average range (except for Vocabulary, *M* = 119). We found significant brain–behavior correlations across groups with verbal IQ, which were largely driven by the group differences (as shown by partial correlation analyses). Therefore, group differences for IQ subtests must be taken into account when interpreting our results.

#### Task order

A possible concern regarding our study relates to the order in which we acquired task-based and resting-state fMRI. Studies found that sometimes resting-state networks are influenced by preceding cognitive activity (Albert et al. 2009; Stevens et al. 2010). Applying this finding to our data, one could be concerned that our resting-state networks could be driven by reading-related tasks participants performed at the beginning of the session. However, the presently found resting-state networks in nonimpaired readers correspond to the resting-state networks identified in other studies. For example, our resting-state network for the left FFG (see Supplementary Fig. 1) shows remarkable consistency with that identified by Vogel et al. (2012; see Fig. 2 on p. 543).

## Conclusion

This study was the first to measure both reading-related and resting-state functional connectivity in dyslexic readers. Our main finding was reduced connectivity in dyslexic readers between left posterior temporal areas (FFG, ITG, MTG, and STG) and the IFG. Reduced connectivity in these networks was consistently present for 2 reading-related tasks and for the resting state, showing a permanent disruption in the functional integration between frontal and temporal brain areas, even present in the absence of explicit task demands and potential group differences in performance.

## Supplementary Material

Supplementary material can be found at: http://www.cercor.oxfordjournals.org/.

## Funding

This work was supported by the Austrian Science Fund: Projects FWF P-23916-B18 and FWF P-18832-B02. F.R. was supported by FWF P-25799-B23 and P.L. was supported by the Doctoral College “Imaging the Mind” (FWF-W1233). The studies were also supported by the European Union (FP6-LIFESCIHEALTH, 18696). Funding to pay the Open Access publication charges for this article was provided by the Austrian Science Fund (FWF P-23916-B18).

## Supplementary Material

Supplementary Data
