# Supplementary material for: Resting-State and Task-Based Functional Brain Connectivity in Developmental Dyslexia
Source: Cereb Cortex. 2014 Aug 28;25(10):3502–14. doi: 10.1093/cercor/bhu184 (PMC4585499; doi:10.1093/cercor/bhu184)
Supplement: Supplementary Data [file supp_25_10_3502__index.html]

Resting-State and Task-Based Functional Brain Connectivity in Developmental Dyslexia — Resting-State and Task-Based Functional Brain Connectivity in Developmental Dyslexia — Resting-State and Task-Based Functional Brain Connectivity in Developmental Dyslexia — Supplementary Data 

# Resting-State and Task-Based Functional Brain Connectivity in Developmental Dyslexia

## Supplementary Data

Supplementary Data

**Files in this Data Supplement:**

- Supplementary Data - Pdf file
