# Supplementary material for: Resting-State and Task-Based Functional Brain Connectivity in Developmental Dyslexia
Source: Cereb Cortex. 2014 Aug 28;25(10):3502–14. doi: 10.1093/cercor/bhu184 (PMC4585499; doi:10.1093/cercor/bhu184)
Supplement: Supplementary Data [file supp_bhu184_bhu184supp.pdf]

Supplementary Figures 1-6. Functional connectivity maps of nonimpaired (left) and dyslexic (middle) readers, and maps of groups differences (right). Maps are separately shown for the resting state, silent reading and the phonological lexical decision. Maps are shown at voxel-wise threshold of p < .001 uncorrected and a cluster extent threshold of $p < .05$ corrected.

Supplementary Table. Reports correlations between brain connectivity estimates (standardized correlation coefficients) and behavioral measures. Details same as Table 3 in the main text.

Functional connectivity of the left fusiform gyrus
x = -46, y = -50, z = -16

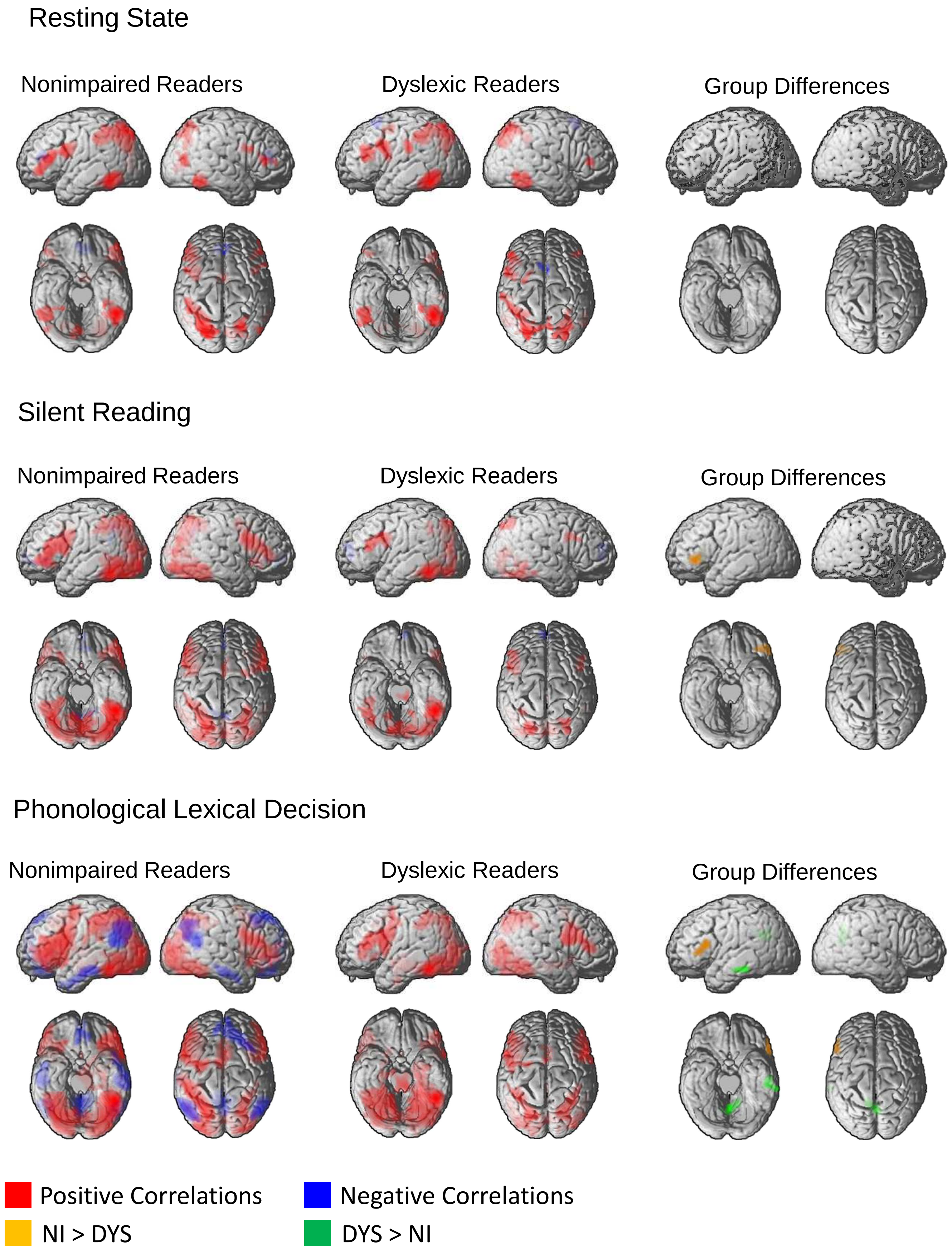

Functional connectivity of the left inferior temporal gyrus
x = -52, y = -62, z = -8

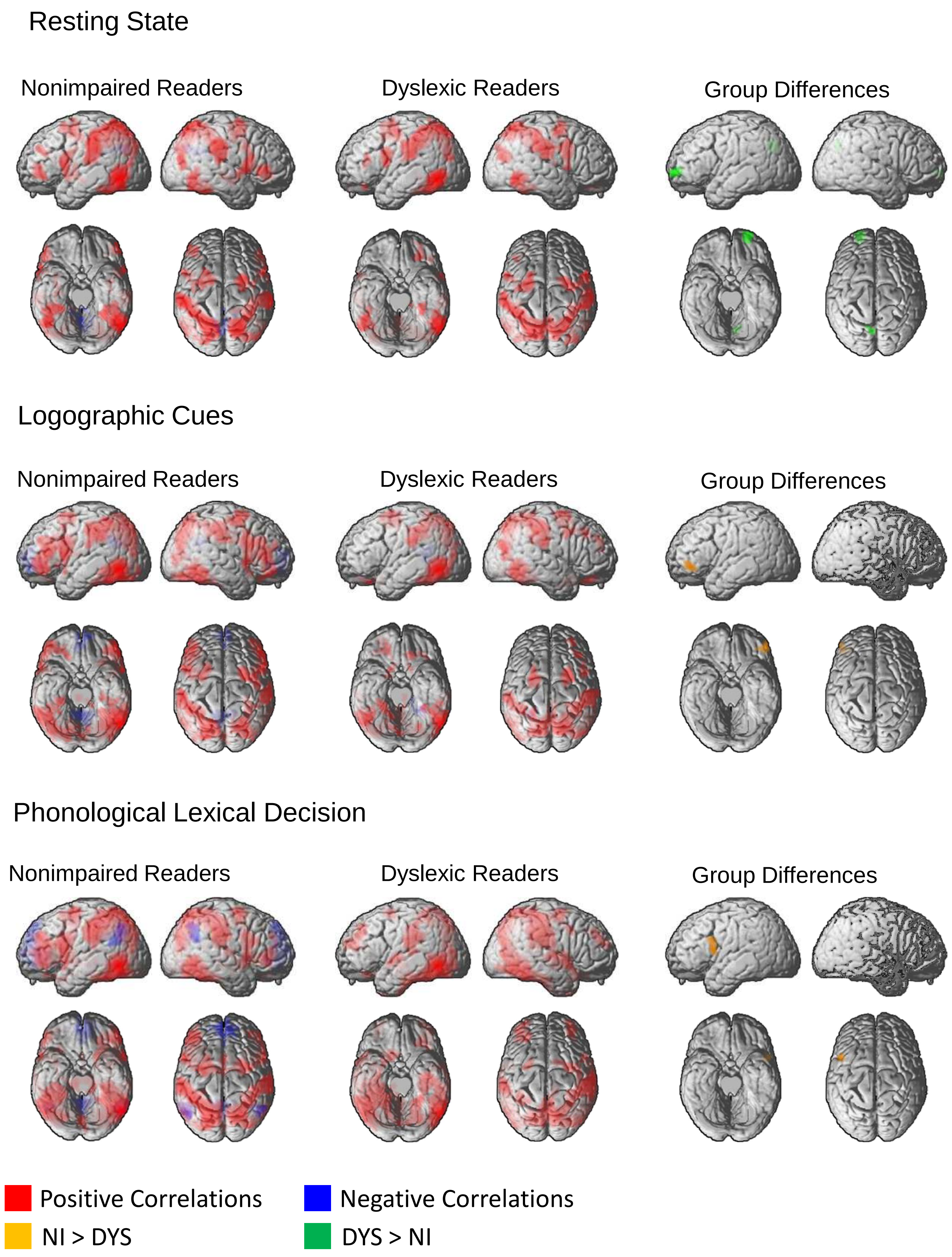

Functional connectivity of the left middle temporal gyrus
x = -60, y = -56, z = 2

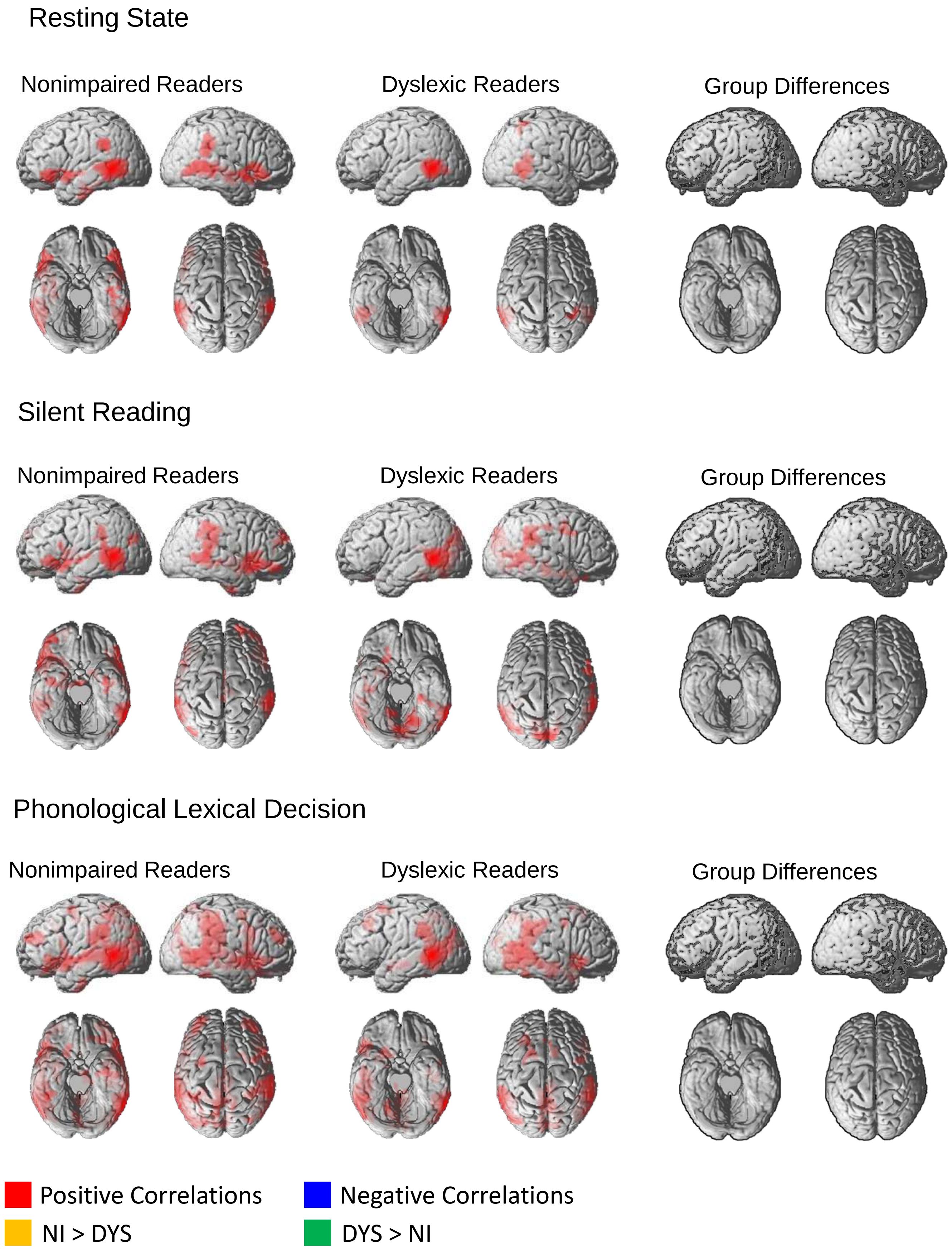

Functional connectivity of the left inferior frontal gyrus
x = -46, y = 16, z = 6

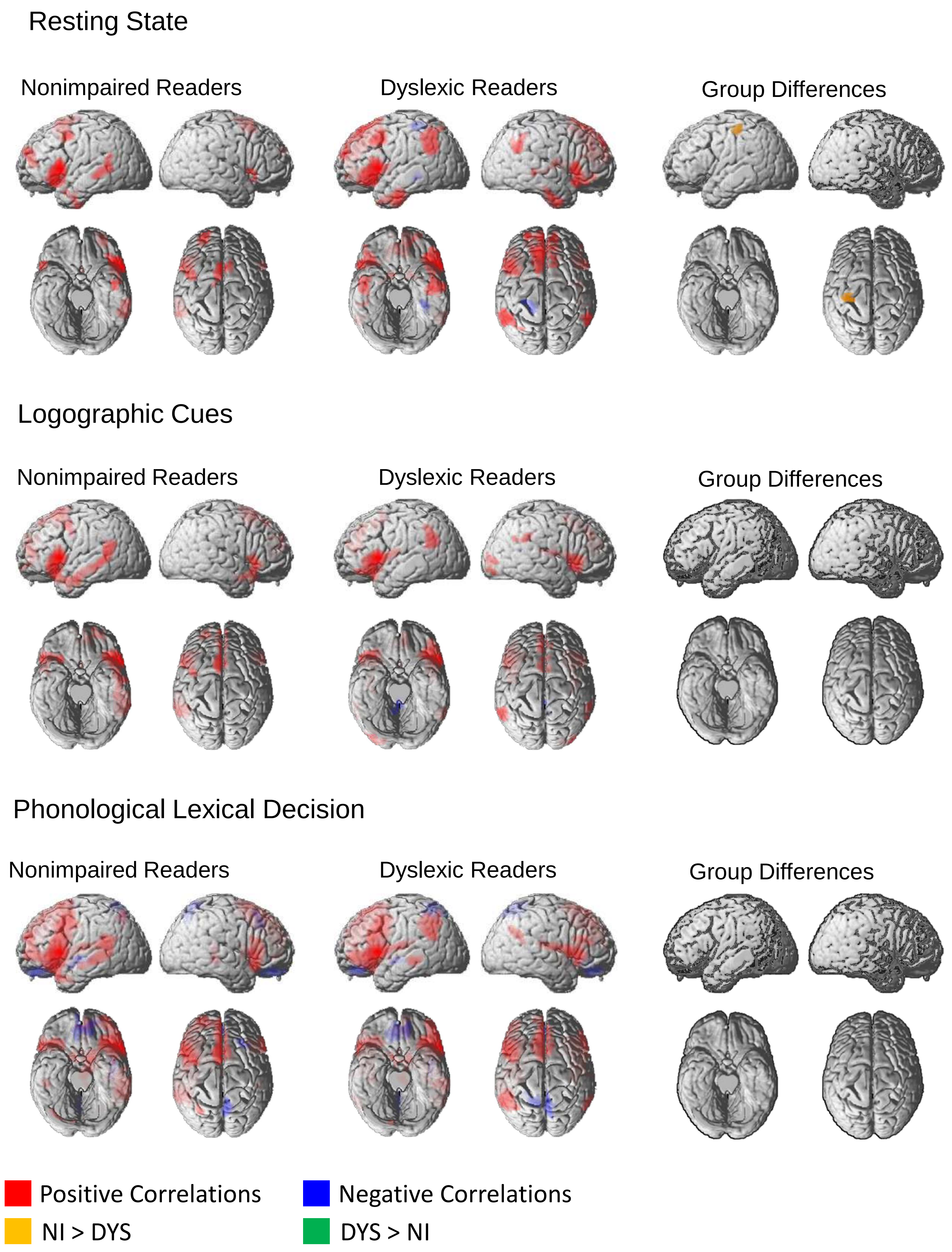

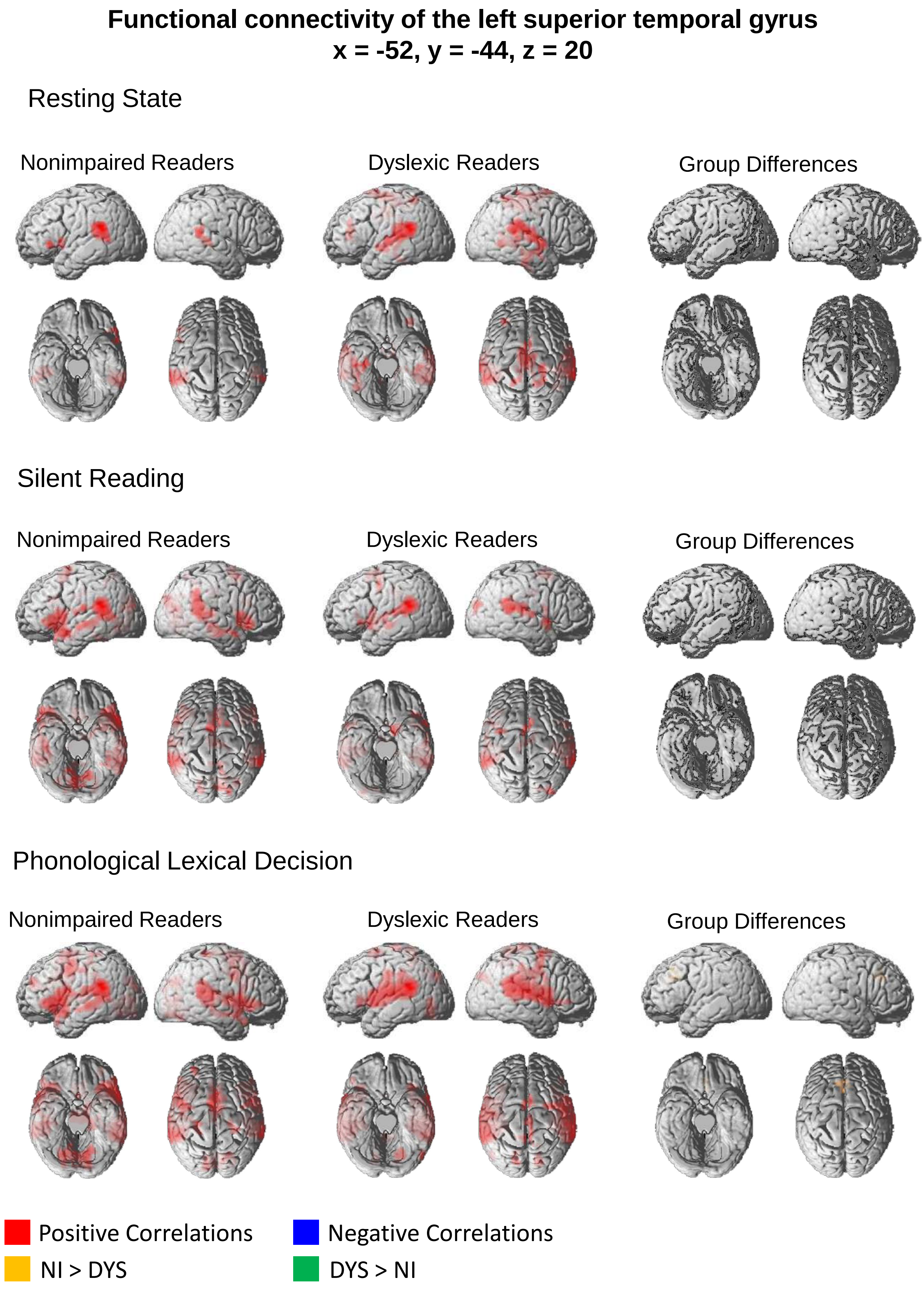
Functional connectivity of the left superior temporal gyrus
x = -52, y = -44, z = 20
Resting State
Nonimpaired Readers
Dyslexic Readers
Group Differences
Silent Reading
Nonimpaired Readers
Dyslexic Readers
Group Differences
Phonological Lexical Decision
Nonimpaired Readers
Dyslexic Readers
Group Differences
Positive Correlations
Negative Correlations
NI > DYS
DYS > NI

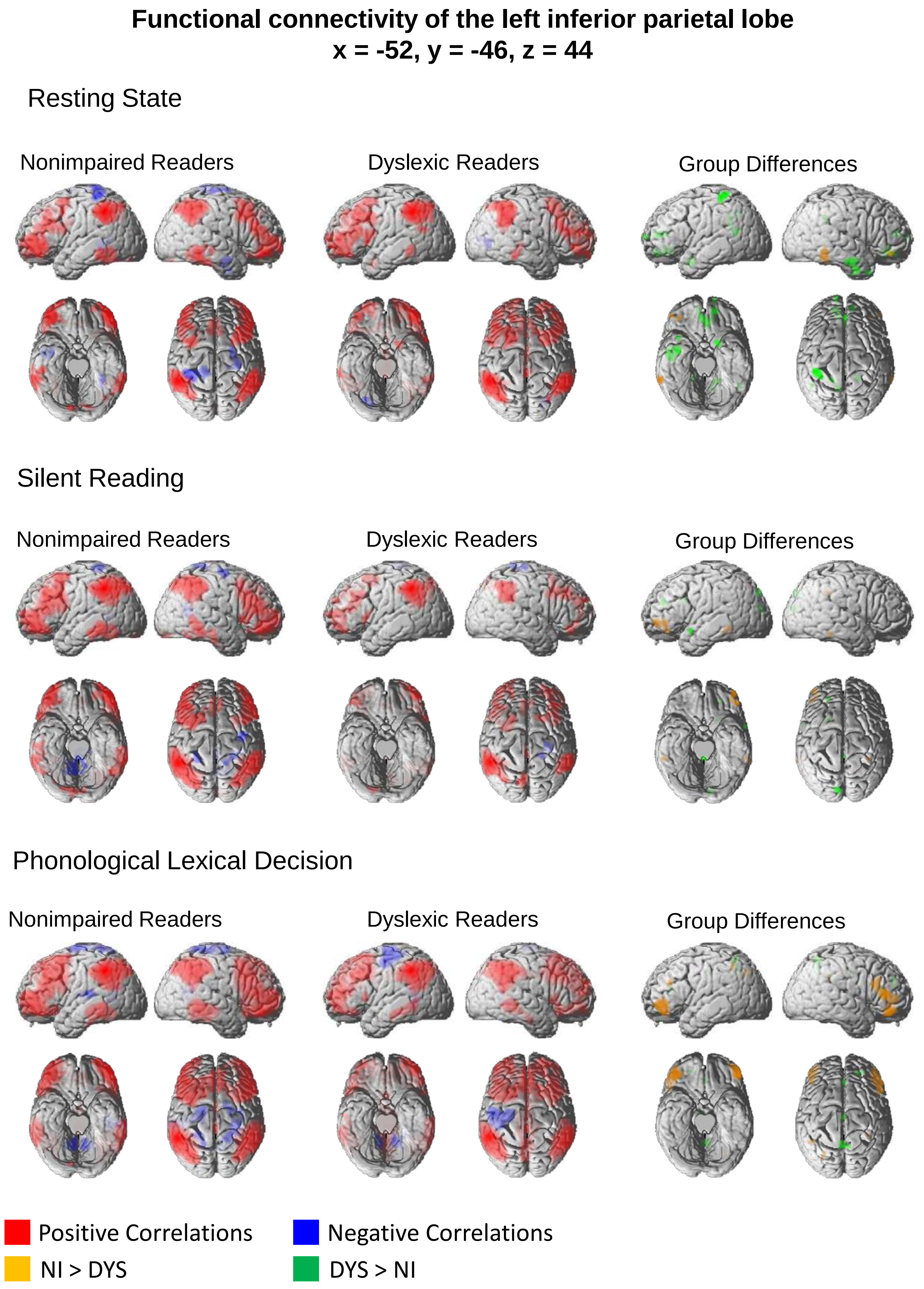
Functional connectivity of the left inferior parietal lobe
x = -52, y = -46, z = 44
Resting State
Nonimpaired Readers
Dyslexic Readers
Group Differences
Silent Reading
Nonimpaired Readers
Dyslexic Readers
Group Differences
Phonological Lexical Decision
Nonimpaired Readers
Dyslexic Readers
Group Differences
Positive Correlations
Negative Correlations
NI > DYS
DYS > NI

Supplementary Table. Brain-behavior correlations (pearson's *r*) for left hemispheric reading related connections.

Connection		Reading	Naming	Verbal IQ
FFG ⬄ L Cereb./OT				
	Rest	.16	.28	.06
	Silent reading	.66***	.42*	.46*
	Phon. Lex. Dec.	.58**	.37	.71***†
FFG ⬄ L IFG tr.				
	Rest	.55**	.27	.53**
	Silent reading	.62**	.28	.40*
	Phon. Lex. Dec.	.48**	.26	.49**
FFG ⬄ R precuneus				
	Rest	-.20	-.08	-.04
	Silent reading	-.49**	-.52**	-.42*
	Phon. Lex. Dec.	-.57**	-.48**	-.57**
ITG ⬄ L OT				
	Rest	.43*	.06	.33
	Silent reading	.65***	.47*	.53**
	Phon. Lex. Dec.	.28	.09	.25
ITG ⬄ L IFG op.				
	Rest	.56**	.48*	.29
	Silent reading	.64***	.57**†	.47*
	Phon. Lex. Dec.	.65***	.38*	.44*
ITG ⬄ L Prec.				
	Rest	-.48*	-.40*	-.50**
	Silent reading	-.32	-.36	-.41*
	Phon. Lex. Dec.	-.14	-.19	-.29
ITG ⬄ R IFG tr.				
	Rest	.57**	.51**	.52**
	Silent reading	.50**	.27	.47**
	Phon. Lex. Dec.	.67***†	.13	.52**
ITG ⬄ L IFG tr.				
	Rest	.54**	.11	.42*
	Silent reading	.57**	.21	.35
	Phon. Lex. Dec.	.59**	.15	.43*
MTG ⬄ L IFG orb.				
	Rest	59**	.18	.19
	Silent reading	.54**	.31	.16
	Phon. Lex. Dec.	.18	.22	-.23†
MTG ⬄ R MFG				
	Rest	.47*	.17	.49*
	Silent reading	.51**	.11	.09
	Phon. Lex. Dec.	.23	-.05	.10
MTG ⬄ L Calc.				
	Rest	-.59**	-.31	-.46*
	Silent reading	-.58**	-.18	-.35
	Phon. Lex. Dec.	-.03	.04	-.04
MTG ⬄ L IFG op.				
	Rest	.41*	.15	.38
	Silent reading	.45*	.34	.37*
	Phon. Lex. Dec.	.35	.10	.20
STG ⬄ L IFG tr.				
	Rest	.55**	.31	.57**
	Silent reading	.65***	.33	.49**
	Phon. Lex. Dec.	.47*	-.13	.20

Supplementary Table. Continued.

IPL ⇔ L MFG orb.				
	Rest	.50**	.32	.52**
	Silent reading	.58**†	.37*	.42*
	Phon. Lex. Dec.	.54**	.22	.40*
IPL ⇔ R IFG orb.				
	Rest	.52**	.29	.44*
	Silent reading	.45*	.14	.50**
	Phon. Lex. Dec.	.71**	.26	.66**
IPL ⇔ R ITG				
	Rest	.61**	.59**	.64***
	Silent reading	.30	.31†	.24
	Phon. Lex. Dec.	.39*	.16	.48**
IPL ⇔ R Hipp.				
	Rest	-.58**	-.30	-.41*
	Silent reading	-.37*	.10†	-.14
	Phon. Lex. Dec.	-.38*	-.29	-.36
IPL ⇔ L Prec.				
	Rest	-.51**	-.27	-.39*
	Silent reading	-.47**	-.24	-.30
	Phon. Lex. Dec.	-.54**	-.15	-.48*
IPL ⇔ L ACC				
	Rest	-.34	-.21	-.13
	Silent reading	-.56**	-.43*	-.66***†
	Phon. Lex. Dec.	-.50**	-.05	-.53**
IPL ⇔ L Post. Cent.				
	Rest	-.48*	-.18	-.48*
	Silent reading	-.01†	-.10	.08
	Phon. Lex. Dec.	.29	.07	.12
IFG ⇔ L MTG peak				
	Rest	.55**†	.49*	.46*
	Silent reading	.69***	.22	.57**
	Phon. Lex. Dec.	.49**	-.09	.30
IFG ⇔ L MTG subcl.				
	Rest	.45*	.22	.33
	Silent reading	.33	-.06	.07
	Phon. Lex. Dec.	.30	.09	-.05†
IFG ⇔ SMA				
	Rest	.37	.19	.38
	Silent reading	.48**	.39*	.29
	Phon. Lex. Dec.	.48*	.20	.44*
IFG ⇔ R rol. operc.				
	Rest	-.35	-.13	-.19
	Silent reading	-.49**	-.14	-.45*
	Phon. Lex. Dec.	-.57**	-.47*	-.54**
IFG ⇔ R AG.				
	Rest	-.55**	-.53**	-.49*
	Silent reading	-.28	-.35	-.25
	Phon. Lex. Dec.	-.39*	-.46*	-.31
IFG ⇔ L IPL/AG				
	Rest	-.55**	-.51**	-.47*
	Silent reading	-.31	-.29	-.32
	Phon. Lex. Dec.	-.39*	-.28	-.11

* *p* <.05, ** *p* <.01, *** *p* <.001, † *p* <.05 in partial correlation controlling for group factor.
